# Induction of Redox-Active Gene Expression by CoCl_2_ Ameliorates Oxidative Stress-Mediated Injury of Murine Auditory Cells

**DOI:** 10.3390/antiox8090399

**Published:** 2019-09-16

**Authors:** Jhang Ho Pak, Junyeong Yi, Sujin Ryu, In Ki Kim, Jung-Woong Kim, Haeri Baek, Jong Woo Chung

**Affiliations:** 1 Department of Convergence Medicine, University of Ulsan College of Medicine and Asan Institute for Life Sciences, Asan Medical Center, 88 Olympic-ro 43-gil, Songpa-Gu, Seoul 05505, Korea; ik.kim@amc.seoul.kr; 2 Department of Otolaryngology—Head and Neck Surgery, University of Ulsan College of Medicine, Asan Medical Center, 88 Olympic-ro 43-gil, Songpa-Gu, Seoul 05505, Korea; junyi0403@gmail.com (J.Y.); lovedbtnwls@naver.com (S.R.); haeribaek@gmail.com (H.B.); 3 Department of Life Science, College of Natural Sciences, Chung-Ang University, 84 Heuksuk-ro, Dongjak-Gu, Seoul 06974, Korea; jwkim0508@gmail.com

**Keywords:** noise-induced hearing loss, oxidative stress, hypoxic preconditioning, HIF-1α, Nrf-2, Prdx6

## Abstract

Free radicals formed in the inner ear in response to high-intensity noise, are regarded as detrimental factors for noise-induced hearing loss (NIHL). We reported previously that intraperitoneal injection of cobalt chloride attenuated the loss of sensory hair cells and NIHL in mice. The present study was designed to understand the preconditioning effect of CoCl_2_ on oxidative stress-mediated cytotoxicity. Treatment of auditory cells with CoCl_2_ promoted cell proliferation, with increases in the expressions of two redox-active transcription factors (hypoxia-inducible factor 1α, HIF-1α, nuclear factor erythroid 2-related factor 2; Nrf-2) and an antioxidant enzyme (peroxiredoxin 6, Prdx6). Hydrogen peroxide treatment resulted in the induction of cell death and reduction of these protein expressions, reversed by pretreatment with CoCl_2_. Knockdown of HIF-1α or Nrf-2 attenuated the preconditioning effect of CoCl_2_. Luciferase reporter analysis with a Prdx6 promoter revealed transactivation of Prdx6 expression by HIF-1α and Nrf-2. The intense immunoreactivities of HIF-1α, Nrf-2, and Prdx6 in the organ of Corti (OC), spiral ganglion cells (SGC), and stria vascularis (SV) of the cochlea in CoCl_2_-injected mice suggested CoCl_2_-induced activation of HIF-1α, Nrf-2, and Prdx6 in vivo. Therefore, we revealed that the protective effect of CoCl_2_ is achieved through distinctive signaling mechanisms involving HIF-1α, Nrf-2, and Prdx6.

## 1. Introduction

A noise exposure-induced reduction in hearing ability is referred to as noise-induced hearing loss (NIHL). Temporary NIHL is referred to as temporary threshold shift of hearing, and it is characterized by a reduced sensitivity to sound over a wide frequency range that is restored gradually to its original level within a short period of time. However, if the noise is intense or the duration of exposure is long enough, hearing loss might be irreversible, referred to as permanent threshold shift of hearing. The pathological mechanisms behind NIHL can be (1) direct mechanical destruction of the hair cell membranes and supporting structures of the organ of Corti, and (2) intense metabolic activities that lead to increased free radical (reactive oxygen and nitrogen species, ROS/RNS) generation in the inner ear tissue mitochondria. Excess generation of free radicals during and after noise exposure suppresses cochlear blood flow that promotes ischemia with further increase in free radical production and a decrease in blood flow. This positive feedback loop eventually induces both necrotic and apoptotic cell death in the organ of Corti [1,2].

Preconditioning is a non-damaging or minimal damage stress condition that potentiates the protective capability against a later and more injurious damage. Various preconditioning strategies such as hyperthermia and noise itself have been applied to protect the cochlea from potential hearing deficits that might be caused by later noise exposure [3,4,5]. In particular, hypoxic preconditioning (8% O_2_ for 4 h) confers significant protection against broadband noise administered 24–48 h after preconditioning in CBA mice. This inner ear resistance to noise injury is associated with increased expression of hypoxia-inducible factor-1α (HIF-1α) within the organ of Corti [6]. It was reported previously that pretreatment with CoCl_2_ under normoxic conditions mimics hypoxic preconditioning and upregulates HIF-1 expression that subsequently confers tolerance to severe hypoxic injury in chick embryos [7]. Additionally, it has been reported that in vivo administration of CoCl_2_ preconditions the mouse heart against global ischemia-reperfusion injury through the activation of HIF-1α, activator protein-1, and inducible nitric oxide synthase [8]. Induction of HIF-1α in inner ears of CoCl_2_-preconditioned mice inhibits noise-induced damage to the inner ear and leads to better recovery of hearing after noise exposure compared to its counterparts [9]. However, the downstream pathway of HIF-1α–mediated preconditioning effect on inner ear remains to be elucidated.

Peroxiredoxins (Prdxs) belong to a superfamily of nonseleno and thiol-dependent peroxidases, catalyzing the reduction of H_2_O_2_, short-chain hydroperoxides, and peroxinitrite. They are widely distributed throughout all kingdoms and are classified as 1/2-cys Prdxs according to the number of conserved catalytic cysteine residues [10]. Of the six mammalian Prdxs, the only 1-cys enzyme is Prdx6 that exhibits a bifunctional enzyme with both peroxidase and Ca^+2^-independent phospholipase A_2_ (aiPLA_2_) activities. Prdx6 has unique characteristics that distinguish it from the other Prdx family members. Notably, it utilizes glutathione (GSH) instead of thioredoxin as a physiological reductant, forms heterodimerization with glutathione S-transferase π (GSTπ) to complete its catalytic cycle, and reduces/hydrolyzes phospholipid hydroperoxides [11]. Prdx6 plays an important role in oxidative stress response as its overexpression in lung carcinoma cells (NCI-H441) reduced cellular OH^-^ levels, and attenuated membrane phospholipid peroxidation and apoptosis induced by Cu^2+^-ascorbate treatment [12]. Meanwhile, antisense-mediated decrease in Prdx6 expression resulted in the accumulation of lipid peroxidation products in the plasma membrane with subsequent apoptotic cell death [13]. The antioxidant protective function of Prdx6 has been further supported by mouse model phenotypes. For example, transgenic Prdx6-overexpressing mice exhibited increased resistance to hyperoxia-induced lung injury [14], while Prdx6-null mice were more susceptible to lung damage with paraquat administration, resulting in increased mortality [15]. Multiple analyses of the Prdx6 promoter region have shown binding sites for various putative regulatory elements [16], Pax5 [17], and several redox-active transcription factors [18,19,20], suggesting that interior and exterior factors such as a change in redox milieu contribute to its transcriptional expression. We have reported recently that retinoic acid-induced Prdx6 expression is involved in rapid hearing recovery after temporary noise exposure. Its transactivation is mediated by a retinoic acid response element on the Prdx6 promoter [21].

In the present study, we examined the protective mechanism behind hypoxia-mediated preconditioning against oxidative burst. Pretreatment of inner ear sensory hair cells with CoCl_2_ resulted in the activation of HIF-1α and Nrf-2 that function as positive transcription factors of Prdx6 gene expression. Additionally, we aimed to determine the immunolocalization of HIF-1α, Nrf-2, and Prdx6 proteins in the cochlear tissues of CoCl_2_-administrated mice.

## 2. Materials and Methods

### 2.1. Materials

All cell culture medium components were purchased from Life Technologies (Gaithersburg, MD, USA) unless otherwise stated. The primary antibodies used in the present study are polyclonal rabbit-anti-Nrf-2 (sc-722), goat-anti-Keap1 (sc-15246), and monoclonal mouse anti-Lamin B (sc-374015) (Santa Cruz Biotechnology, Santa Cruz, CA, USA), polyclonal rabbit anti-HIF-1α, (ab2185, Abcam, Cambridge, MA, USA), polyclonal rabbit anti-Prdx6 (LF-PA0011) and rabbit anti-glyceraldehyde-3-phosphate dehydrogenase (GAPDH, LF-PA0018), AbFrontier Co., Seoul, Korea). Horseradish peroxidase (HRP)-conjugated secondary antibodies were purchased from Jackson ImmunoResearch Laboratories Inc. (West Grove, PA, USA). An immortalized auditory cell line (HEI-OC1) derived from the organ of Corti of Immortomouse transgenic mice was kindly provided by Dr. Federico Kalinec (Dept. of Cell and Molecular Biology, House Ear Institute, Los Angeles, CA, USA). All other chemicals (biotechnology grade) were purchased from Sigma-Aldrich (St. Louis, MO, USA).

### 2.2. Cell Culture and CoCl_2_/H_2_O_2_ Treatment

**Figure 1:**
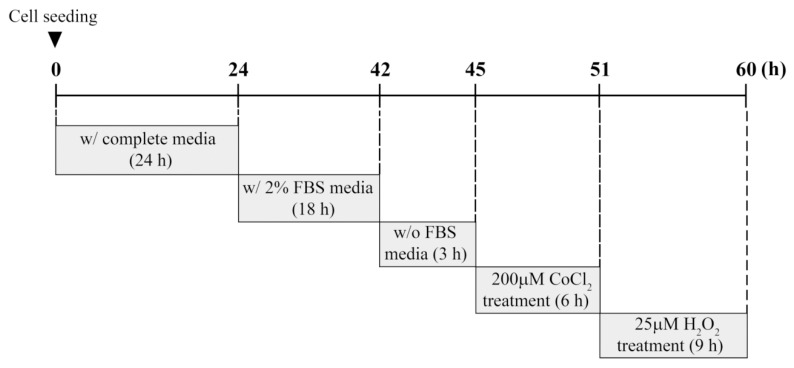
Experimental scheme for preconditioning with CoCl_2_ followed by H_2_O_2_ treatment.

The establishment and characterization of the immortalized HEI-OC1 auditory cells were performed as described previously [22]. HEI-OC1 cells were cultured in high-glucose Dulbecco’s modified Eagle’s medium containing 10% fetal bovine serum (FBS) and penicillin (100 U/mL)/streptomycin (100 μg/mL) at 33 °C with 10% CO_2_ atmosphere in a humidified incubator. For experiments involving CoCl_2_ or H_2_O_2_ exposure, cells were seeded at ~70% confluence on 60-mm culture dishes and cultured for 24 h under standard conditions. Cells were deprived gradually of serum by incubation in 2% FBS overnight, followed by incubation in serum-free medium for 3 h. These serum-starved cells were exposed to different concentrations of CoCl_2_ or H_2_O_2_ at the indicated times. In preconditioning studies, cells were preincubated with 200 μM CoCl_2_ for 6 h. Next, the culture medium containing CoCl_2_ was changed, and cells were treated with 25 μM H_2_O_2_ for 9 h. This experimental procedure including time schedule is summarized in Figure 1.

### 2.3. Cytotoxicity Assay

Cell viability was measured by a Cell Counting Kit-8 (CCK-8, Dojindo Laboratories, Kumamoto, Japan) according to the manufacturer’s instructions. The cells were seeded onto 96-well plates at a density of 5 × 10^3^ cells/well. Following serum starvation, cells were treated with CoCl_2_, H_2_O_2_, or both for 24 h as described above. The amount of dark blue formazan product was determined by measuring absorbance at 450 nm using a microplate spectrophotometer (Molecular Devices Corp., Sunnyvale, CA, USA). The absorbance value in untreated control cells was taken as 100% of viability.

### 2.4. Detection of Intracellular Oxygen Radicals

Intracellular ROS level was measured using a fluorescent dye, 5-(and-6)-chloromethyl-2′,7′-dichlorodihydrofluorescein diacetate acetylester (CM-H_2_DCFDA, Molecular Probes, Inc., Eugene, OR, USA). Serum-starved cells grown on 96-well plates (4 × 10^3^ cells/well) were treated with 200 μM CoCl_2_ for 6 h, 25 μM H_2_O_2_ for 9 h, or both as described above. Cells were then washed twice with Hank’s balanced salt solution (HBSS) and incubated with 5 μM CM-H_2_DCFDA at 33 °C for 20 min in the dark. After washing with HBSS, the levels of DCF fluorescence were immediately measured using a luminescence spectrofluorometer (VICTOR 3; Perkin-Elmer, Waltham, MA, USA) with excitation and emission wavelengths of 485 and 535 nm, respectively. The values were converted to folds for comparison with the untreated control.

### 2.5. Construction of Murine NRF-2 or HIF-1α Gene Knockdown (KD) Plasmids

The shRNA expression vectors targeting the murine Nrf-2 and HIF-1α genes were generated by designing target DNA oligonucleotides (Table 1). The expression vectors were subcloned into pLKO.1-puro lentiviral vector (Addgene, Cambridge, MA, USA) double-digested with Age I and EcoR I (pLKO-mNrf-2 or pLKO-mHIF-1α). Subsequently, each recombinant plasmid was transformed into competent DH5α *E. coli* cells. Correct short hairpin (sh) sequence formation was confirmed by DNA sequencing.

### 2.6. Construction of ARE- and HRE-Deleted Reporter Plasmids (pGL3-mPxΔARE or pGL3-mPxΔHRE)

A luciferase reporter plasmid (pGL3-mPx) containing the proximal 5′-flanking region of the murine Prdx6 promoter (668bp) has been described elsewhere [17,21]. In each mutant-reporter plasmid, the deletion of the HRE or antioxidant response element (ARE) consensus sequences within the Prdx6 promoter was done with the QuikChange site-directed mutagenesis kit (Stratagene, La Jolla, CA, USA) using pGL3-mPx as the template and appropriate sets of primers. DNA sequencing was used to verify the mutant constructs with the deletions (pGL3-mPxΔARE or pGL3-mPxΔHRE).

### 2.7. Transfection of NRF-2 or HIF-1α KD Plasmids into HEI-OC1 Cells

HEI-OC1 cells that reached ~70% confluence, were transfected with the reporter plasmids using Lipofectamine 2000 (Invitrogen, Carlsbad, CA, USA). HEI-OC1 cells were transfected with the same amount of mock vector as an internal control. Forty-eight hours after transfection, cells were harvested for immunoblot analysis of Nrf-2 or HIF-1α expression. To evaluate the cell viability, each transfectant was treated with CoCl_2_, H_2_O_2_, or both as described above.

### 2.8. Luciferase Reporter Assay

HEI-OC1 cells were seeded in 24-well culture plates at a density of 1 × 10^5^ cells/well and cotransfected with pGL3-mPx and each shRNA expression vector (pLKO-mNrf-2 or pLKO-mHIF-1α). Additionally, cells were cotransfected with wild- or mutant-reporter plasmids (pGL3-mPxΔARE or pGL3-mPxΔHRE) and Nrf-2 or HIF-1α overexpression plasmids (pcDNA-Nrf-2 or HA-HIF-1α-pcDNA3) as described elsewhere [20]. For the normalization of transfection efficiency, the pCMV-β-gal plasmid was simultaneously transfected into the cells. About 48 h after transfection, cells were treated with CoCl_2_ (200 μM) for 6 h. Luciferase and β-galactosidase activities from total cell lysate were measured according to respective Bright-Glo Luciferase and Beta-Glo Assay kits (Promega, Madison, WI, USA) using a microplate luminometer (Perkin-Elmer). The luciferase activities of individual reporter plasmids were normalized to those of β-galactosidase.

### 2.9. Immunoblot Analysis

Cells were washed with ice-cold PBS and lysed with RIPA buffer (Sigma-Aldrich) supplemented with complete protease inhibitor cocktail and centrifuged at 13,000× *g* at 4 °C for 20 min. Nuclear proteins were isolated using the NE-PER Cytoplasmic and Nuclear Protein extraction kit (Pierce Biotechnology, Rockford, IL, USA) according to the manufacturer’s instructions. Protein concentration was determined with the BCA Protein Assay kit (Pierce Biotechnology). Next, 30 μg of total soluble proteins or 5 μg of nuclear proteins were separated by SDS-PAGE and transferred to nitrocellulose membranes (Merck-Millipore, Billerica, MA, USA). The membranes were probed with primary antibodies (1:1000 dilution) described in Materials, followed by incubation with appropriate secondary antibodies (1:5000 dilution). The immunoreactive bands were visualized with a West-Q chemiluminescent Substrate kit (GenDEPOT, Barker, TX, USA) and the band intensities on films were analyzed by densitometry to quantify protein expression using a FluorS MultiImager (Bio-Rad, Hercules, CA, USA). The membranes then were washed with Restore Western Blot Stripping Buffer (Thermo Scientitis, Waltham, MA, USA) and reprobed with 1:1000 diluted anti-GAPDH polyclonal or anti-Lamin B polyclonal antibodies to normalize for cytosolic or nuclear protein loading, respectively.

### 2.10. Administration of Mice with CoCl_2_

All experimental procedures were performed in compliance with the guidelines of the National Institutes of Health and the Declaration of Helsinki. The Committee on the Use and Care of Animals of the University of Ulsan approved protocols. Animal care was performed under the supervision of the Laboratory Animal Unit of the Asan Institute for Life Sciences (IACUC No. 2016-13-271; date of approval, November 14, 2016). Male CBA mice at 5–6 weeks (~20 g) of age (Oriental Charles River Technology, Seoul, Korea) were intraperitoneally administered with vehicle (saline) or CoCl_2_ (60 mg/kg body weight). After 6 h, both cochleae were removed, fixed with 4% formaldehyde/1% glutaraldehyde in 0.1 M sodium phosphate buffer, decalcified in 5.5% EDTA, and embedded in paraffin.

### 2.11. Immunohistochemistry

Deparaffinized and rehydrated cochlear paraffin sections (5-μm thick) were heated in a microwave oven in 10 mM sodium citrate buffer (pH 6.0) for antigen retrieval and then pretreated with 3% H_2_O_2_ in 0.1 M Tris-buffered saline (TBS) (pH 7.4) to quench endogenous peroxidase activity. The sections were incubated with TBS containing 5% normal goat serum and the primary antibodies (1:500 dilution for HIF-1α and Nrf-2, 1:7500 dilution for Prdx6) overnight at 4 °C, followed by goat anti-rabbit polyclonal HRP-conjugated secondary antibody (1:100 dilution; DakoNorth America, Inc., Carpinteria, CA, USA). Immunostaining of each protein was visualized with an ImmPACT™ DAB Peroxidase Substrate kit (Vector Laboratories, Burlingame, CA, USA). The sections were counterstained with Mayer’s hematoxylin, dehydrated, cleared in xylene, and mounted in Permount. Images of sections were recorded with an upright microscope (Nikon Eclipse Ci, Tokyo, Japan).

### 2.12. Statistical Analysis

Data were expressed as means ± standard deviation (SD) of three or more independent experiments. Differences between groups were evaluated using the Student *t*-test or two-way ANOVA with Tukey’s post-hoc test, as appropriate. Differences between mean values were considered statistically significant at *p* < 0.05.

## 3. Results

### 3.1. Effects of CoCl_2_ on Cell Viability and HIF-1α, Nrf-2, and Prdx6 Protein Expression

**Figure 2:**
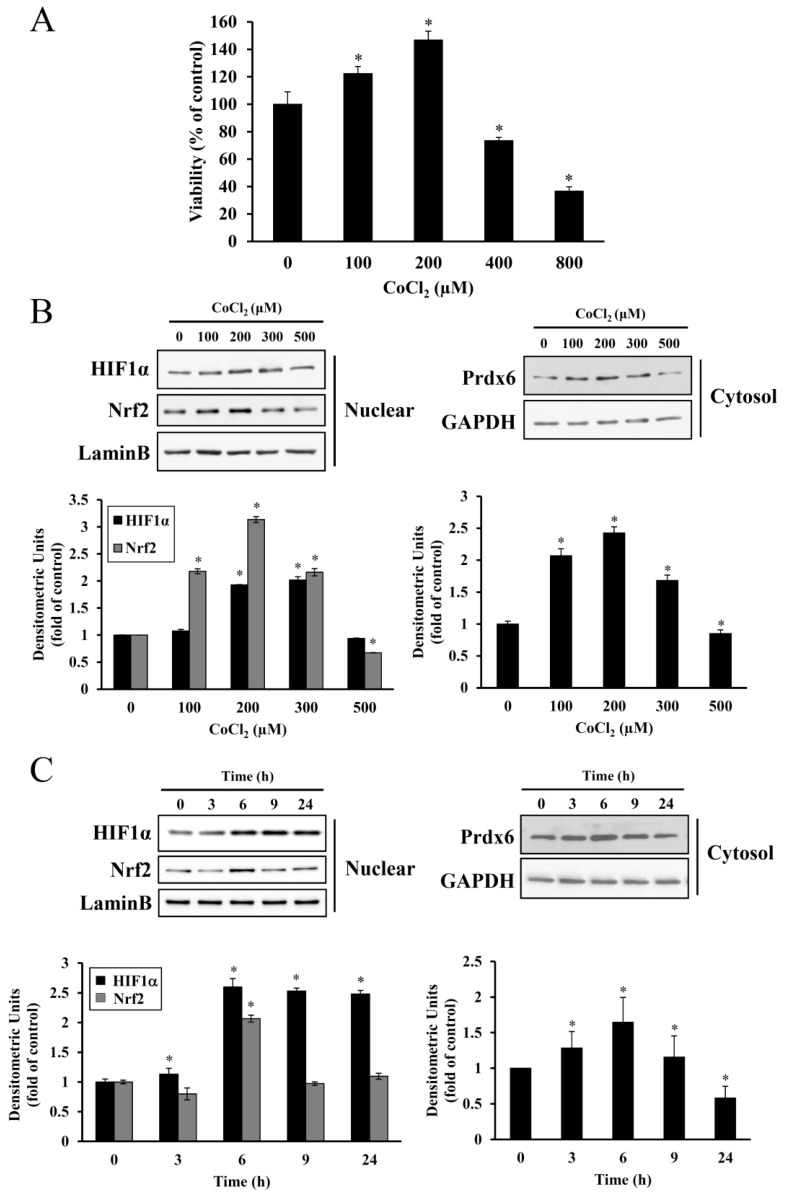
Effect of CoCl_2_ on HEI-OC1 cell proliferation and HIF-1α, NRF-2, and *Prdx6* expression. (**A**) Cells were treated with 0–800 μM CoCl_2_ for 6 h, replaced with fresh medium, and further cultured for 24 h. Cell proliferation was then determined using a CCK-8 assay, which measures the production of formazan dye by live cells. Data are presented as means ± SDs for three independent experiments, expressed as a percentage of the untreated control. * *p* < 0.05, compared with untreated control. (**B**,**C**) After treatment with 0–500 μM CoCl_2_ for 6 h or 200 μM CoCl_2_ for the indicated times, nuclear or cytosolic proteins were immunoblotted for HIF-1α, Nrf-2, and Prdx6 expression, respectively. The membranes were then stripped and reprobed with Lamin B or GAPDH polyclonal antibody (a control for protein loading). Individual data were quantified as densitometric units and normalized with expected loading control proteins. Each data represents fold changes relative to untreated controls or the zero time point. Data are presented as means ± SDs for the three independent experiments (* *p* < 0.05, compared with untreated control or zero time point control).

We reported previously that hypoxic preconditioning mediated by CoCl_2_ injection prevented hearing loss in noise-exposed mice [9]. To know whether this preconditioning effect was applicable to auditory cells, serum-starved HEI-OC1 cells were exposed to 100–800 μM CoCl_2_ for 6 h, followed by incubation in serum-free medium for 24 h. The CCK-8 assay showed that CoCl_2_-induced cell proliferation increased in a dose-dependent manner up to 200 μM. Higher concentration treatment (400–800 μM) resulted in the reduction of cell viability, indicating that relatively low concentrations of CoCl_2_ (i.e., mild hypoxic condition) was required for cell proliferation (Figure 2A).

It has been well known that hypoxic conditions not only result in the upregulation of HIF-1α and its target genes but also ROS generation. Therefore, we examined whether the expressions of HIF-1α, another redox-active transcription factor, and target genes such as NRF-2 and Prdx6 were regulated by CoCl_2_ exposure. Immunoblot analysis revealed nuclear accumulation of HIF-1α and Nrf-2 at 100–300 μM of CoCl_2_, concomitant with increased *Prdx6* expression. At 500 μM, the expression levels of HIF-1α and Nrf-2 decreased to nearly similar levels with those of untreated cells or below basal levels (Figure 2B). Since treatment of HEI-OC1 cells with 200 μM CoCl_2_ for 6 h triggered the highest proliferation and maximum increase in HIF-1α, NRF-2, and Prdx6 (Figure 2C) expression, the same CoCl_2_ concentration, and duration of exposure were used in subsequent preconditioning studies.

### 3.2. Cytotoxic Effect of H_2_O_2_ on HEI-OC1 Cells

**Figure 3:**
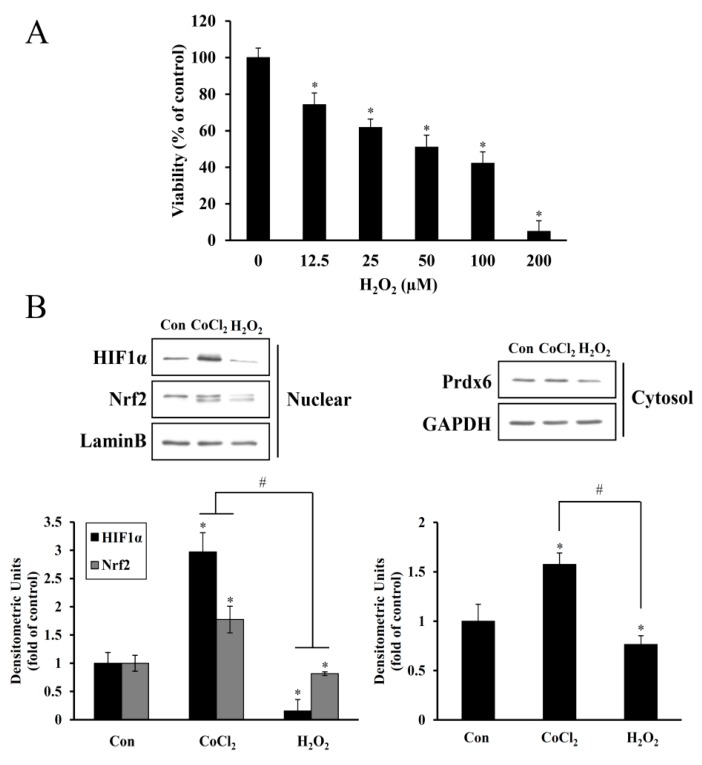
Inhibitory effect of H_2_O_2_ on HEI-OC1 cell viability and HIF-1α, NRF-2, and Prdx6 expression. (**A**) After exposure to 0–200 μM H_2_O_2_ for 24 h, cell viability was determined using the CCK-8 assay. Data are presented as means ± SDs for the three independent experiments, expressed as a percentage of the untreated control. * *p* < 0.05, compared with the untreated control. (**B**) Cells treated with 200 μM CoCl_2_ for 6 h or 100 μM H_2_O_2_ for 24 h were harvested, and nuclear and cytosolic proteins were analyzed by immunoblotting for HIF-1α, Nrf-2, and Prdx6. Protein bands were quantified using densitometry, and their abundances were expressed relative to the density of Lamin B or GAPDH band. The ratio of HIF-1α and Nrf-2 to Lamin B or Prdx6 to GAPDH are presented as fold changes relative to the untreated control. Data are presented as the means ± SDs of three independent experiments (* *p* < 0.05, compared with the control; ^#^ *p* < 0.05, CoCl_2_ versus H_2_O_2_).

HEI-OC1 cells were exposed to 12.5–200 μM of H_2_O_2_ for 24 h. Next, cell survival rates were examined by the CCK-8 assay. As Figure 3A shows, H_2_O_2_ treatment decreased the viability of cells in a dose-dependent manner, in which its significant reduction was evident at concentrations of 12.5 μM and higher, relative to that of an untreated control. Additionally, the expression levels of HIF-1α, Nrf-2, and Prdx6 proteins were inversely proportional to dose-dependent cytotoxicity (data not shown), suggesting that the expression of these proteins is indispensable for cell survival after exposure to oxidative stress. Indeed, while 6 h of CoCl_2_ (200 μM) treatment resulted in significantly elevated expression levels for these proteins, 24 h of H_2_O_2_ (100 μM) treatment resulted in dramatically reduced expression levels to below that of an untreated control (Figure 3B). Their self-inductions were detected as early as 3 h of the exposure and began to decline gradually at 6 h (Figure S1).

### 3.3. Effect of CoCl_2_ Preconditioning on H_2_O_2_-Induced Cytotoxicity

**Figure 4:**
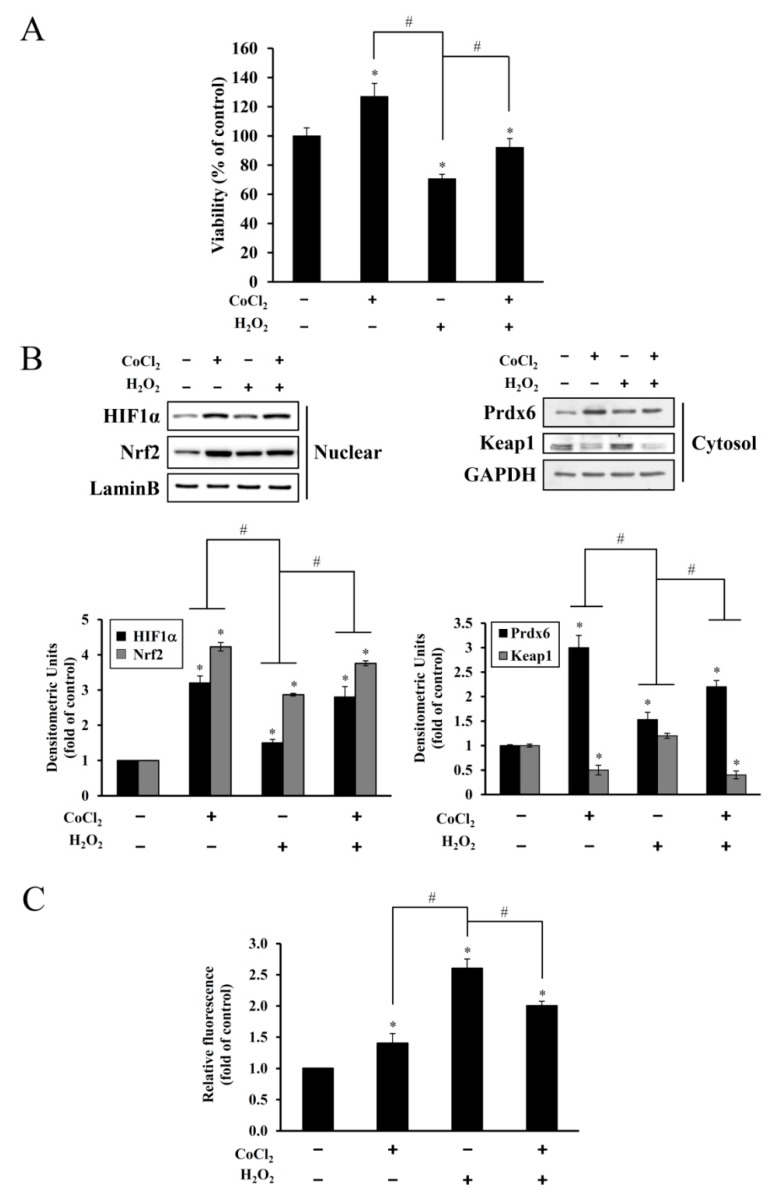
Protective effect of CoCl_2_ on H_2_O_2_-induced cytotoxicity, repression of HIF-1α, Nrf-2, Keap1, and Prdx6 expression, and ROS accumulation. HEI-OC1 cells were preincubated with 200 μM CoCl_2_ for 6 h before exposure to 25 μM H_2_O_2_ for 9 h. (**A**) The CCK-8 assay was used to determine cell viability. Data are presented as means ± SDs for three independent experiments expressed as a percentage of the untreated control (* *p* < 0.05, compared with the untreated control, ^#^ *p* < 0.05, CoCl_2_ only versus H_2_O_2_ only or CoCl_2_ plus H_2_O_2_). (**B**) Representative immunoblot showing HIF-1α, Nrf-2, Keap1, and Prdx6 expression. Individual bands were quantified densitometrically and normalized to Lamin B (HIF-1α and Nrf-2) or GAPDH (Prdx6 and Keap1). Values in the graphs are presented as fold changes relative to the untreated control, expressed as means ± SDs of three independent experiments (* *p* < 0.05, compared with the untreated control, ^#^ *p* < 0.05, CoCl_2_ only versus H_2_O_2_ only or CoCl_2_ plus H_2_O_2_). (**C**) After the treatment as described above, the levels of ROS were determined by measuring CM-H_2_DCFDA. Data are presented as fold changes relative to the untreated control, expressed as ± SDs of three independent experiments (* *p* < 0.05, compared with the untreated control, ^#^ *p* < 0.05, CoCl_2_ only versus H_2_O_2_ only or CoCl_2_ plus H_2_O_2_).

To examine the preconditioning effect of CoCl_2_ on H_2_O_2_-exposed cells, HEI-OC1 cells were first preincubated with 200 μM CoCl_2_ for 6 h followed by incubation with 25 μM H_2_O_2_ for 9 h. The CCK-8 assay was used to assess cell viability. As Figure 4A shows, pretreatment with CoCl_2_ improved cell viability by ~25% regarding that observed for H_2_O_2_ exposure alone. Concomitant with this, CoCl_2_ pretreatment resulted in the recovery of HIF-1α and Nrf-2 nuclear accumulation and Prdx6 expression, as well as degradation of cytosolic Keap1 (Figure 4B), suggesting that the maintenance of the expression levels of these proteins is closely associated with cell survival against oxidative insults.

Next, CoCl_2_ treatment resulted in increased generation of intracellular ROS, and further increases in H_2_O_2_-treated cells by ~2.6-fold. Pretreatment of CoCl_2_ significantly reduced ROS accumulation mediated by H_2_O_2_, but still higher than that of an untreated control (Figure 4C). Taken together, these results suggest that a relatively moderate level of ROS may induce the activities of antioxidant enzymes such as HIF-1α, Nrf-2, and Prdx6, preventing oxidative cytotoxicity.

### 3.4. Inhibitory Effect of HIF-1α or NRF-2 knockdown (KD) on Prdx6 Expression and Preconditioning

**Figure 5:**
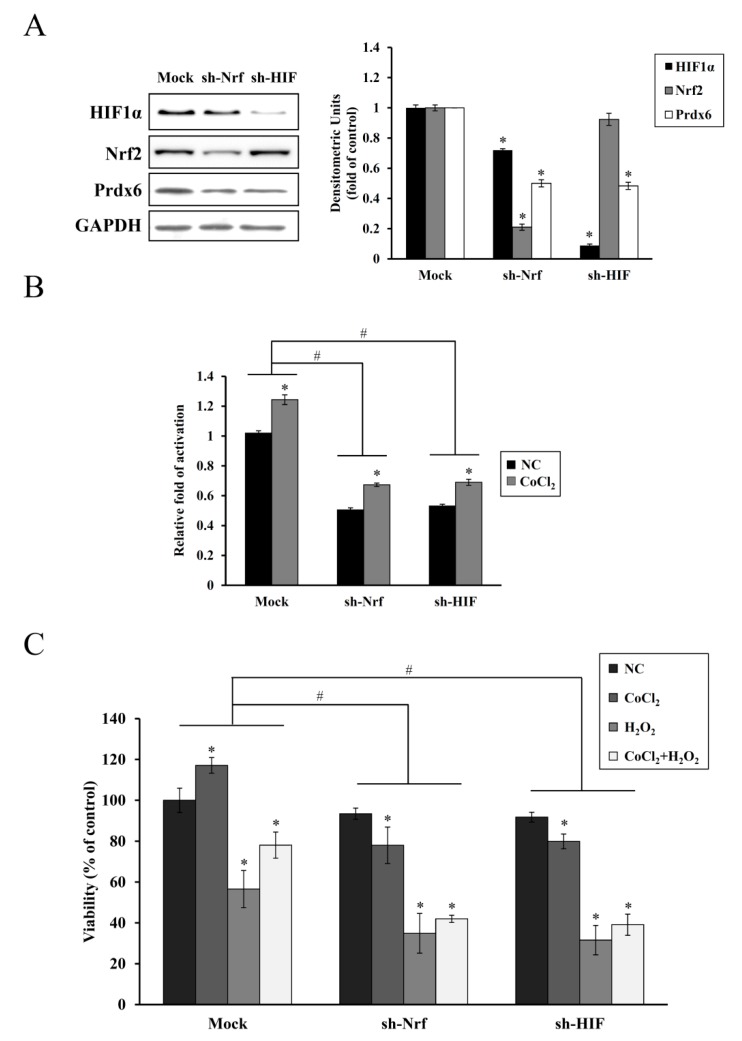
Inhibitory effect of Nrf-2 and HIF-1α knockdown on Prdx6 expression and preconditioning induced by CoCl_2_. (**A**) HEI-OC1 cells transfected with the pLKO.1(mock), pLKO-mNrf-2 (sh-Nrf), or pLKO-mHIF-1α (sh-HIF) were treated with 200 μM CoCl_2_ for 6 h. Next, Nrf-2, HIF-1α, and Prdx6 expression were analyzed via immunoblotting. Individual bands were quantified densitometrically and normalized to GAPDH. Values in the graph are presented as fold changes relative to the mock transfectant, expressed as means ± SDs of three independent experiments (* *p* < 0.05, compared with the mock control) (**B**) Cells were cotransfected with the pGL3-mPx luciferase reporter vector and each KD plus the β-galactosidase plasmid. After 24 h, cells were treated with 200 μM CoCl_2_ for 6 h, and luciferase activities were determined. Luciferase activities were normalized to that of β-galactosidase. Data are presented as means ± SDs for three independent experiments, expressed as a fold change relative to the mock transfectant. * *p* < 0.05, compared with the untreated control (NC), ^#^ *p* < 0.05, mock group versus sh-Nrf or sh-HIF group. (**C**) Cells transfected with mock or each KD plasmid were treated with 200 μM CoCl_2_ for 6 h, 25 μM H_2_O_2_ for 9 h, or CoCl_2_ plus H_2_O_2_, and cell viability was evaluated using the CCK-8 assay. Data are expressed as a percentage of the mock/untreated control and presented as means ± SDs for three independent experiments. * *p* < 0.05, compared with the untreated control (NC), ^#^ *p* < 0.05, mock group versus sh-Nrf or sh-HIF group.

Since HIF-1α and Nrf-2 are known transcription factors for the *Prdx6* gene [20], we examined whether changes in HIF-1α or NRF-2 expression levels had any effect on *Prdx6* expression. Cells were transiently transfected with the HIF-1α and Nrf-2 KD plasmids, or an empty vector. At 48 h after transfection, cells were exposed to 200 μM CoCl_2_ for a further 6 h. Immunoblot analysis revealed ~0.8- or ~0.9-fold reduced expression of Nrf-2 or HIF-1α in HIF-1α and Nrf-2 KD cells treated with CoCl_2_ compared to their expression levels in mock plasmid transfected cells. Additionally, the level of Prdx6 expression in both KD transfectants was markedly decreased by ~0.5-fold. While Nrf-2 KD suppressed the expression of HIF-1α by ~0.25-fold, HIF-1α KD did not affect the level of Nrf-2 expression (Figure 5A). Cotransfection of pGL3-mPx reporter with each KD plasmid resulted in a ~0.6-fold decrease in *Prdx6* promoter-driven luciferase activity induced by CoCl_2_ (Figure 5B), suggesting that both HIF-1α and Nrf-2 function as positive transcription factors for *Prdx6*. Relative luciferase activity of each KD transfectants was further decreased in the absence of CoCl_2_, probably due to the silence of residual HIF-1α or NRF-2 gene that was escaped from KD. Moreover, protective CoCl_2_-mediated preconditioning effect on H_2_O_2_-induced cytotoxicity was markedly attenuated in each KD transfectants relative to that of mock transfectants (Figure 5C). A lesser but significant reduction of cell viability by Nrf-2 or HIF-1α KD was observed in each untreated transfectant, indicating that their basal expressions are required for cell survival.

### 3.5. Transactivation of the Prdx6 Promoter by HIF-1α and Nrf-2

**Figure 6:**
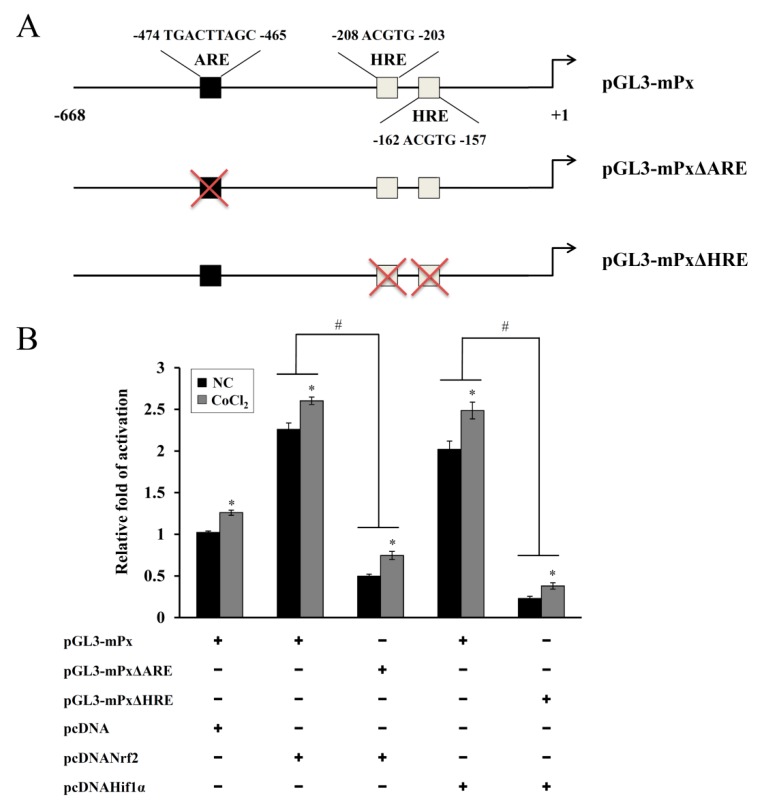
Role of the HRE and ARE in Prdx6 gene expression. (**A**) Schematic illustration of the Prdx6 promoter containing ARE and two HRE sites (a black box for Nrf-2 binding site, white boxes for HIF-1α binding sites). Deletion of the ARE or two HREs were generated by site-directed mutagenesis of the full-length promoter (pGL3-mPx) in the pGL3-Basic vector, namely pGL3-mPxΔARE and pGL3-mPxΔHRE. (**B**) Each reporter vector and the β-galactosidase expression plasmid were cotransfected with the empty pcDNA3 vector, the Nrf-2, or HIF-1α expression plasmid into HEI-OC1 cells. After 48 h, cells were incubated with 200 μM CoCl_2_ for 6 h, and the luciferase activity was determined. Luciferase activities were normalized to that of β-galactosidase. Data are presented as the means ± SDs of three independent experiments. * *p* < 0.05, compared with the untreated control (NC), ^#^ *p* < 0.05, pGL3-mPx group versus pGL3-mPxΔARE or pGL3-mPxΔHRE group.

To evaluate the direct correlation between the expression of HIF-1α, Nrf-2, and *Prdx6* genes, we analyzed the binding sites for HIF-1α and Nrf-2 in the murine *Prdx6* promoter. A web-based computer analysis (Matlnspector; Genomatrix) revealed the presence of two putative HIF-1α binding sites on hypoxic response elements (HRE) at positions, −208 to −203 and −162 to −157, and validated Nrf-2 binding to the ARE between −474 and −465 (Figure 6A) described elsewhere [23]. To verify that the regions responsible for HIF-1α- and Nrf-2-mediated activation were HRE and ARE respectively, reporter constructs with deleted HRE or ARE regions were tested in cotransfection experiments with the HIF-1α or Nrf-2 overexpression plasmids with or without CoCl_2_ treatment. Generation of reporter *HIF-1α* or *NRF-2* overexpression plasmids using the wild-type reporter (WT, pGL3mPx) markedly elevated the luciferase activity even in the absence of CoCl_2_ compared with that in the mock transfectant, demonstrating their roles in positive transcription factors of *Prdx6* gene expression. The CoCl_2_-driven luciferase activity was further elevated by ~1.2-fold. On the other hand, this activity was significantly suppressed in ARE or HRE deletion constructs (pGL3-mPxΔARE or pGL3-mPxΔHRE) even after CoCl_2_ induction and exogenous overexpression of HIF-1α or Nrf-2 (Figure 6B), indicating that both ARE and HRE are indispensable for Prdx6 promoter activity.

### 3.6. Distribution of HIF-1α, NRF-2, and Prdx6 Expression in Mouse Cochleae

**Figure 7:**
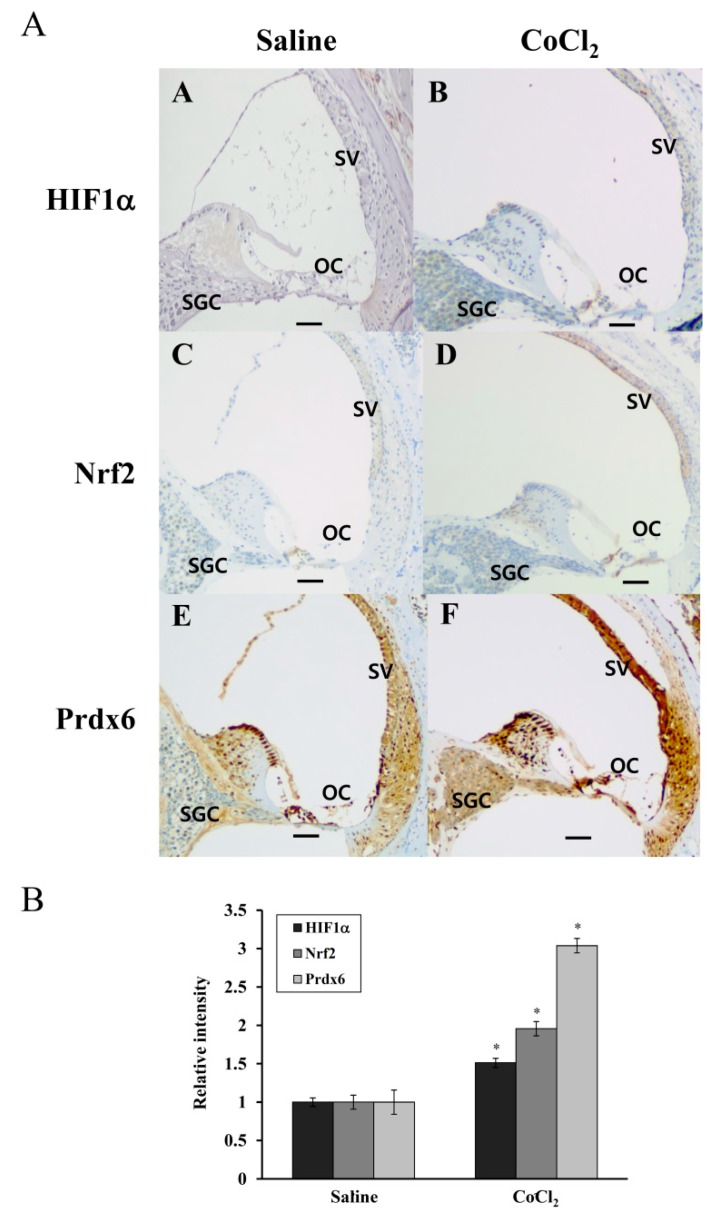
HIF-1α, Nrf-2, and Prdx6 expression in the cochleae of saline- or CoCl_2_-injected mice. Mouse cochlear paraffin sections were used for immunohistochemical analyses of HIF-1α, Nrf-2, and Prdx6 expression. (**A**) The expressions of HIF-1α (A: saline-injected, B: CoCl_2_-injected), Nrf-2 (C: saline-injected, D: CoCl_2_-injected), and Prdx6 (E: saline-injected, F: CoCl_2_-injected) are marked with brown-colored deposits and nuclei are counterstained with hematoxylin. OC, organ of Corti, SV, stria vascularis; SGC, spiral ganglion cells. Original magnification ×100, scale bar = 100 μm. (**B**) The intensities of the dark brown dots in images were quantified using an Image J program. Data in the graph is presented as fold changes relative to the saline-injected cochleae, expressed as ± SDs of three independent experiments. * *p* < 0.05, compared with the saline-injected cochleae.

To know whether CoCl_2_-induced upregulation of HIF-1α, Nrf-2, and Prdx6 proteins occurred in vivo, we immunohistochemically examined their expressions in the cochlea tissues from mice intraperitoneally injected with saline or CoCl_2_. As Figure 7A shows, the expressions of HIF-1α and Nrf-2 were observed more intensely in the organ of Corti (OC), spiral ganglion cells (SGC), and stria vascularis (SV) of CoCl_2_-injected mice than those of saline-injected ones. Immunoreactive expression of Prdx6 in these regions was much stronger in CoCl_2_-injected mice. Overall increases in HIF1α, Nrf-2, and Prdx6 expression in CoCl_2_-injected cochlea were ~1.3-, ~2-, and ~3.3-fold, respectively, compared with saline-injected one (Figure 7B). These results indicating that elevated expression of Prdx6 is closely correlated with a CoCl_2_-induced increase in the protein expression levels of HIF-1α and Nrf-2 in the cochlear tissues.

## 4. Discussion

It is well established that a noise exposure induced elevation in free radical generation in the cochlea causes oxidative stress-induced sensory hair cell damage with subsequent acoustic trauma such as NIHL. Since the regeneration of injured auditory neurons and hair cells is difficult, there is no effective treatment after the development of a permanent threshold shift. In this respect, the paradigm of preconditioning is one of the most effective approaches for intrinsic protection of the cochlea against noise-induced damage. We reported previously the protective effect of intraperitoneal CoCl_2_ preinjection on sensory hair cell loss and hearing impairment induced by white band noise exposure in mice [9]. In the present study, we found that CoCl_2_ pretreatment protected auditory hair cells (HEI-OC1) from H_2_O_2_-mediated cytotoxicity via the activation of redox-sensitive transcription factors HIF-1α and Nrf-2 and their target gene Prdx6, demonstrating an in vitro hypoxic preconditioning mechanism for noise-induced cochlear cell injury.

Preconditioning with mild hypoxia can protect various tissues against subsequent lethal hypoxic/ischemic injuries. In the cochlea, sublethal ischemia prevented lethal ischemia-induced hair cell degeneration and ameliorated hearing impairment, suggesting ischemic tolerance [24]. Systemic hypoxia in mice resulted in smaller permanent threshold shifts at all tested frequencies, compared to the air-exposed control [6]. An appealing mechanism for hypoxia-mediated protection is the elevated antioxidative capacity regulation of later excessive free radical formation within the cochlea and other inner ear structures during and after noise exposure. Hypoxic preconditioning can be chemically mimicked using CoCl_2_, where ionized cobalt interferes with intracellular oxygen homeostasis. Cobalt is an essential element found in the form of cyanocobalamin (vitamin B_12_) and it is critical for animals due to its involvement in red blood cell production and nervous system function. Although exposure to a high dose or prolonged exposure to a low dose of CoCl_2_ induces apoptosis and necrosis with inflammatory responses, its beneficial effects include stimulation of erythropoiesis and angiogenesis, promotion of tissue adaptation to hypoxia, and improvement of ischemic/hypoxic tolerance [25]. In the present study, treatment with 100–200 μM CoCl_2_ for 6 h resulted in the proliferation of HEI-OC1 cells. However, at higher dose ranges, this proliferative effect was reduced (Figure 2), suggesting a dose and exposure time-dependent optimal preconditioning effect. The same CoCl_2_ concentration range has been reported to promote the proliferation and migration of rhesus choroid endothelial cells [26].

Cobalt triggers free radical generation via a Fenton-like reaction. We observed intracellular ROS generation slightly increased in HEI-OC1 cells exposed to CoCl_2_ for 6 h, as assessed by a peroxide-sensitive fluorescent probe, CM-H_2_DCFDA (Figure 4C). In contrast to the detrimental effects of an abrupt alternation of redox status probably due to massive ROS production, moderate amounts of ROS act as second messengers in signal transduction and gene regulation in various cell types, thus protecting against lethal oxidative stress. HIF-1α and Nrf-2 are well known redox-sensitive transcription factors that activate the expression of genes involved in the adaptation of cells and tissues to hypoxia and detoxification/antioxidant defense, respectively [27]. In the present study, maximal accumulation of HIF-1α and Nrf-2 was detected in the nuclei of HEI-OC1 cells treated with CoCl_2_ for 6 h, and a concurrent maximal elevation was observed in the cytosolic levels of their target, Prdx6 (Figure 2). This exposure time coincided with the highest proliferation rate mediated by CoCl_2_, indicating that ROS generation under these conditions promotes the induction of redox response genes and cellular proliferation.

Excessive free radical generation induced by noise exposure causes the accumulation of lipid peroxidation products, oxidized proteins, and oxidative DNA adducts in the cochlea and other inner ear cells, promoting the death of the hair cells and nerve endings with subsequent hearing loss. Exposure of mice to intense broadband noise resulted in the significant elevation of cochlear ROS levels within 1–2 h following the exposure, along with anatomical abnormalities of the OC and a permanent threshold shift [28]. Additionally, intense noise increased the expression of lipid peroxidation products such as 8-isoprostane in the SV, SGC, and the OC of guinea pigs. In the OC, the heavily immunoreactive outer hair cells than inner hair cells were well correlated with permanent hair cell damage [29]. The accumulation of ROS and RNS markers appeared rapidly and transiently in the inner ear during and following high-intensity noise exposure, while hair cell loss progressively increased over time, stabilizing two or more weeks after a single insult. Therefore, oxidative/nitrative stress triggered by noise begins early and persists for an extended time even after termination of noise exposure [30]. In all, these findings imply that noise-induced immediate hair cell injury might be attributable to mechanical irritation plus transiently intensive free radical generation, whereas its continuous generation contributes to long-term hair cell loss and eventually, permanent hearing loss. In the present study, noise-induced oxidative stress in hair cells was mimicked by direct H_2_O_2_ exposure, causing a dose-dependent increase in cell death and suppression of HIF-1α, NRF-2, and Prdx6 expression to below the expression levels observed in the untreated control (Figure 3). This oxidative stress-mediated cytotoxicity, protein degradation, and intracellular ROS accumulation were attenuated by CoCl_2_ pretreatment (Figure 4). The protective effect of CoCl_2_ on oxidative injury has been also reported in HepG2 tumor cells treated with tert-butyl hydroperoxide/serum deprivation [31], neonatal piglets that underwent hypothermic circulatory arrest [32], and in rats exposed to hypobaric hypoxia-induced oxidative stress [33], where the upregulation of HIF-1α and its target proteins play crucial roles for benefiting from CoCl_2_ preconditioning.

In addition to HIF-1α [6,9], a protective role has been reported for Nrf-2 in NIHL animal models. The Nrf-2/heme oxygenase-1 (HO-1) signaling pathway was activated in the OC of rats intraperitoneally administered with rosmarinic acid, resulting in the reduction of the noise-induced oxidative stress triggered a generation of superoxide and lipid peroxidation [34]. Nrf-2-null mice exhibited more severe impairment of hearing than its WT counterparts at day 7 after noise exposure, whereas treatment with Nrf-2-activating drugs before noise exposure preserved the integrity of hair cells and improved post-exposure hearing levels in only the WT mice. Moreover, a single nucleotide polymorphism in the human NRF-2 promoter was associated with sensory neuronal hearing loss [35]. In the present study, shRNA KD of HIF-1α or NRF-2 abolished the endogenous upregulation of HIF-1α or NRF-2 along with Prdx6 expression and cell proliferation induced by CoCl_2_ pretreatment, thus, failing subsequent cytoprotection against oxidative insults (Figure 5). A similar correlation between reduced HIF-1α and Nrf-2 expression and increased apoptotic cell death has been previously reported in chronic hypoxia-mediated neurodegeneration [36]. In particular, we found that Nrf-2 KD attenuated the induction of HIF-1α expression, suggesting its role as a transcriptional regulator of *HIF-1α* expression. This is consistent with a previous finding, where downregulation of *NRF-2* expression via shRNA interference impaired the hypoxia-mediated induction of HIF-1α and its target gene expression in glioblastoma cells [37]. Nrf-2 is also required for the arsenite-mediated upregulation of HIF-1α in HepG2 hepatoma cells [38]. A direct regulatory connection between Nrf-2 and HIF-1α via the Nrf-2 binding site (ARE) in the enhancer region upstream of HIF-1α gene has been recently reported, indicating that Nrf-2 plays a role in HIF-1α expression [39].

Elevated ROS generation or oxidative stress actively induces Prdx6 expression. Prdx6 functions as an antioxidant through its peroxidase activity, reducing various oxidants including H_2_O_2_, short-chain hydroperoxides, oxidized fatty acids, and phospholipid hydroperoxides [11]. Transcriptional regulation of *Prdx6* gene is mediated by multiple redox-active transcription factors to its promoter [20]. In the present study, one ARE and two HRE consensus sequences were observed in the murine Prdx6 promoter and both Nrf-2 and HIF-1α contributed to the CoCl_2_-induced expression of Prdx6, as confirmed by the KD and mutant promoter/reporter assays (Figure 5 and Figure 6). ChIP analyses using primer sets covering the ARE and HRE regions and a respective antibody revealed that their binding abilities were substantially increased by CoCl_2_ treatment (data not shown), confirming that both sequences are functionally responsible for the binding of Nrf-2 and HIF-1α, respectively. These results indicate that Nrf-2 and HIF-1α recognize their respective ARE and HRE sequences within the murine Prdx6 promoter and function as transactivators for the Prdx6 gene during the CoCl_2_ preconditioning period. Consistent with the findings from the present study, it has been shown in other studies that the transcriptional upregulation of other antioxidants/phase II-detoxifying enzymes such as HO-1 in Kupffer cells from ethanol-fed rats is mediated by the binding of Nrf-2 and HIF-1α to their respective sequences within the HO-1 promoter, thus protecting the liver from alcohol-induced injury [40]. The Keap1/Nrf-2/ARE pathway-mediated upregulation of *Prdx6* mRNA expression has been reported in human lung carcinoma cells (A549) and primary rat alveolar type II cells treated with H_2_O_2_ or tert-butylhydroquinone [19]. Additionally, Prdx6 expression has been associated with cell viability in retinal ganglion cells treated with CoCl_2_, where the prolonged exposure triggered reduction in Prdx6 expression was related to an increase in hypoxia-induced cell death [41]. In all, these findings implicate that an Nrf-2/HIF-1α pathway regulated induction of Prdx6 expression plays protective roles in oxidative stress-induced cell death.

Accumulation of HIF-1α was reported previously in the noise-exposed cochlea of mice, where its expression was elevated in OC, SV, and SGC after a 3h exposure to white band noise with 120dB peak equivalent sound pressure level [42]. Furthermore, the induction of HIF-1α expression by CoCl_2_ preinjection minimized sensory hair cell loss caused by subsequent noise exposure in these regions, compared with the saline preinjected controls [9]. In the human cochlea, Nrf-2 immunoreactivity was detected in the cytoplasm and nuclei of hair and supporting cells of OC and the vestibular sensory epithelia, and its reactivity significantly decreased with age [43]. Consistent with these findings, the present study showed a more intense immunoreactivity for HIF-1α, Nrf-2, and Prdx6 in the OC, SV, and SGC regions of CoCl_2_-injected cochleae compared to that observed in the same regions of saline-injected controls (Figure 7). Considering the accumulation of free radicals in these regions during and after noise exposure and their antioxidant capacities, it is likely that the induction of HIF-1α and Nrf-2 by CoCl_2_ injection leads to the transcriptional upregulation of Prdx6, in turn protecting the cochlea from noise-triggered oxidative damage. Prdx6 is widely distributed throughout all major mammalian organs with high contents in the lung, brain, liver, kidney, and testis. Within tissues, Prdx6 expression is the highest in epithelium, including apical regions of olfactory and respiratory epithelium and skin epidermis, where it functions in vivo as an antioxidant enzyme [11]. Since noise-induced cochlear damage is caused by direct mechanical destruction, excessive free radical generation, and reduction of cochlear blood flow, elevated protein expression of Prdx6 in OC, SV, and SGC postulates that it might be involved in repairing noise-mediated mechanical wounds, antioxidative defense, as well as restoration of redox homeostasis. This is supported by findings from previous studies showing endogenous Prdx6 overexpression in the hyperproliferative epidermis of mouse skin wounds [44] and actively proliferating epithelia and anterior stroma of wounded rat corneas after photorefractive keratectomy [45]. Moreover, Prdx6 has been reported to participate in the protection of the epidermis from UV-induced damage and the formation of new blood vessels in inflamed tissues caused by excision injury, suggesting its requirement for blood vessel integrity and viability in the wounded tissue [46]. To our knowledge, this is the first report of Prdx6 immunolocalization to mouse cochlear tissues.

## 5. Conclusions

In conclusion, we have shown that CoCl_2_ pretreatment before severe oxidative insult attenuates oxidative stress-induced cytotoxicity in auditory hair cells. The protective effect of CoCl_2_ is achieved through the activation of an antioxidant defense signaling pathway involving Nrf-2, HIF-1α, and Prdx6. Our findings improve the knowledge of the beneficial mechanisms behind hypoxic preconditioning, and at the same time provide a new paradigm for the rational design of co-therapies for preventing/repairing cochlear injuries caused by acoustic trauma.

## Figures and Tables

**Table 1:** Murine HIF-1α and Nrf-2 short hairpin (sh) DNA sequences.

Short Hairpin (sh) DNA Sequences (5′ to 3′)		
HIF-1α	F:	CCG GTG GAT AGC GAT ATG GTC AAT GCT CGA GCA TTG ACC ATA TCG CTA TCC ATT TTT G
	R:	AAT TCA AAA ATG GAT AGC GAT ATG GTC AAT GCT CGA GCA TTG ACC ATA TCG CTA TCC A
Nrf-2	F:	CCG GCT TGA AGT CTT CAG CAT GTT ACT CGA GTA ACA TGC TGA AGA CTT CAA GTT TTT G
	R:	AAT TCA AAA ACT TGA AGT CTT CAG CAT GTT ACT CGA GTA ACA TGC TGA AGA CTT CAA

## Supplementary Materials

The following are available online at https://www.mdpi.com/2076-3921/8/9/399/s1, Figure S1: Expression of HIF-1, Nrf2, and Prdx6 at 0–6 h of 100 μM H_2_O_2_ treatment.
