# Supplementary material for: Induction of Redox-Active Gene Expression by CoCl2 Ameliorates Oxidative Stress-Mediated Injury of Murine Auditory Cells
Source: Antioxidants (Basel). 2019 Sep 16;8(9):399. doi: 10.3390/antiox8090399 (PMC6769615; doi:10.3390/antiox8090399)
Supplement: Supplementary file 1 [file antioxidants-08-00399-s001.pdf]

antioxidants

Supplementary Materials.

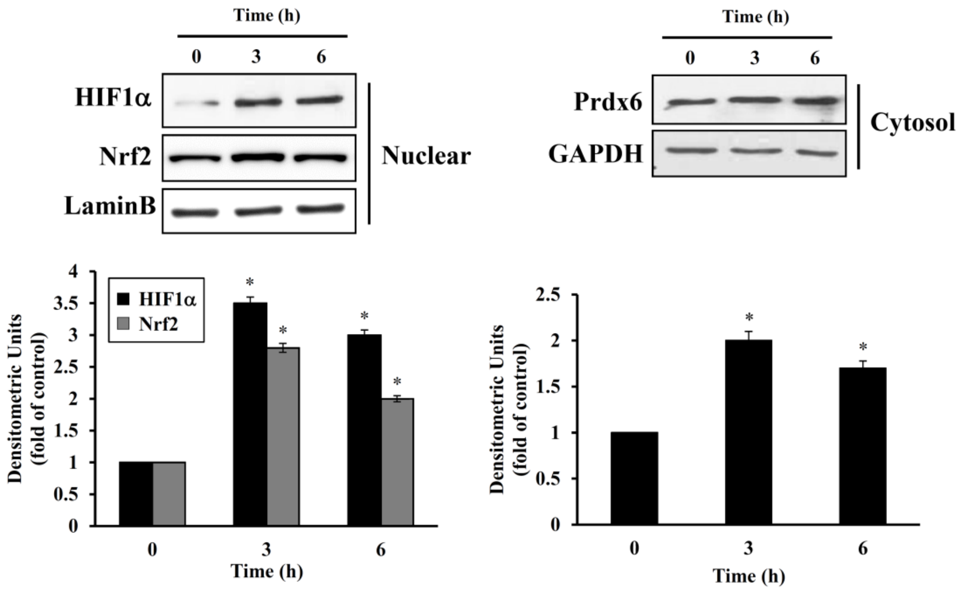

Figure 1. Expression of HIF-1α, Nrf2, and Prdx6 at 0–6 h of 100 μM H_2O_2 treatment. Cells were treated with 100 μM H_2O_2 for the indicated times and nuclear and cytosolic proteins were analyzed by immunoblotting for HIF-1α, Nrf-2, and Prdx6. Protein bands were quantified using densitometry, and their abundances were expressed relative to the density of Lamin B or GAPDH band. The ratio of HIF-1α and Nrf-2 to Lamin B or Prdx6 to GAPDH are presented as fold changes relative to the untreated control. Data are presented as the means ± SDs of three independent experiments (*$p < 0.05$, compared with the control; $^{\#}p < 0.05$, $CoCl_2$ versus H_2O_2).
